# ppGpp, the General Stress Response Alarmone, Is Required for the Expression of the α-Hemolysin Toxin in the Uropathogenic *Escherichia coli* Isolate, J96

**DOI:** 10.3390/ijms232012256

**Published:** 2022-10-14

**Authors:** Jorge Fernández-Vázquez, Juan David Cabrer-Panes, Anna Åberg, Antonio Juárez, Cristina Madrid, Tania Gaviria-Cantin, Llorenç Fernández-Coll, Andrés Felipe Vargas-Sinisterra, Carlos Jonay Jiménez, Carlos Balsalobre

**Affiliations:** 1 Department of Genetics, Microbiology and Statistics, School of Biology, University of Barcelona, 08028 Barcelona, Spain; 2 Department of Medical Biochemistry and Biophysics, Umeå University, SE-90187 Umeå, Sweden; 3 Institute for Bioengineering of Catalonia, The Barcelona Institute of Science and Technology, 08028 Barcelona, Spain

**Keywords:** α-hemolysin, ppGpp, UPEC, gene regulation

## Abstract

ppGpp is an intracellular sensor that, in response to different types of stress, coordinates the rearrangement of the gene expression pattern of bacteria to promote adaptation and survival to new environmental conditions. First described to modulate metabolic adaptive responses, ppGpp modulates the expression of genes belonging to very diverse functional categories. In *Escherichia coli*, ppGpp regulates the expression of cellular factors that are important during urinary tract infections. Here, we characterize the role of this alarmone in the regulation of the *hlyCABD_II_* operon of the UPEC isolate J96, encoding the toxin α-hemolysin that induces cytotoxicity during infection of bladder epithelial cells. ppGpp is required for the expression of the α-hemolysin encoded in *hlyCABD_II_* by stimulating its transcriptional expression. Prototrophy suppressor mutations in a ppGpp-deficient strain restore the α-hemolysin expression from this operon to wild-type levels, confirming the requirement of ppGpp for its expression. ppGpp stimulates *hlyCABD_II_* expression independently of RpoS, RfaH, Zur, and H-NS. The expression of *hlyCABD_II_* is promoted at 37 °C and at low osmolarity. ppGpp is required for the thermoregulation but not for the osmoregulation of the *hlyCABD_II_* operon. Studies in both commensal and UPEC isolates demonstrate that no UPEC specific factor is strictly required for the ppGpp-mediated regulation described. Our data further support the role of ppGpp participating in the coordinated regulation of the expression of bacterial factors required during infection.

## 1. Introduction

In order to survive and colonize specific niches within their hosts, bacterial pathogens must rapidly respond to environmental signals and adjust the expression pattern of cellular factors involved in its pathogenesis. For this purpose, regulatory systems that control and coordinate the expression of functionally related subsets of genes are required. Chemically diverse molecules, such as proteins, regulatory RNAs, and low molecular weight non-proteinaceous molecules, also known as alarmones, can act as regulators of those regulatory circuits. The level of the alarmones can be quickly altered by specific enzymatic activities, thus mediating rapid changes in the pattern of gene expression. Guanosine tetra- and pentaphosphate, hereafter referred to as ppGpp, are modified nucleotides that are synthesized by the bacterial cells in response to different environmental signals as a result of unfavorable growth conditions [1,2]. In *Escherichia coli*, ppGpp turnover depends on two enzymes: the ribosome-associated RelA synthetase and the SpoT protein, which has both synthetase and hydrolase activities [1]. In *E. coli*, under standard laboratory conditions, ppGpp levels are low during exponential growth (40 µM), increasing about 20-fold upon entry into stationary phase and later stabilizing at 150 M [3]. ppGpp, initially described as a mediator of the global metabolic response to amino acid starvation (the stringent response), is also involved in the regulation of many other cellular functions related with the adaptation of bacterial cells to environmental stresses [1,4,5,6]. Hence, it has been described that ppGpp controls the expression of genes involved in the ability of bacterial pathogens to colonize their hosts [7,8,9].

Uropathogenic *E. coli* (UPEC) causes urinary tract infections (UTIs). UPEC expresses several adhesins enabling bacterial cells to colonize different parts of the urinary tract and, often, UPEC also produces toxins such as the α-hemolysin and the cytotoxic necrotizing factor [10].

The secreted α-hemolysin plays a key role during UTI by causing cytolytic and cytotoxic effects in a wide range of mammalian cell types [11,12,13]. The α-hemolysin belongs to the family of RTX toxins characterized by some conserved structural features and by sharing a common organization of their genetic determinants. The *hlyCABD* operon codes for genes that enable bacterial cells to synthesize, activate, and secrete the α-hemolysin [14]. The precursor of the active toxin (HlyA, molecular mass 110 kDa), is activated in the cytoplasm by HlyC, a fatty acid acyltransferase [15]. The active α-hemolysin is directly secreted from the cytoplasm to the extracellular milieu through a transmembrane channel consisting of three proteins, namely, HlyB, HlyD, and TolC [16]. The UPEC isolate J96, isolated from a pyelonephritis patient, encodes two *hlyCABD* operons, denoted I and II, located within two independent pathogenicity islands [17,18]. The *hlyCABD_II_* operon has been extensively studied and was used in fundamental studies demonstrating the existence of virulence factors in bacteria [11].

In previous reports, it was demonstrated that ppGpp plays a crucial role in the control of the expression of important colonization factors during the establishment of UTI by UPEC, such as type 1 fimbriae, antigen 43, and flagella [19,20,21]. Here, we found that this alarmone controls also the expression of the *hlyCABD_II_* operon. The amount of α-hemolysin secreted and its associated cytotoxicity is importantly diminished in a strain deficient in ppGpp production. Our data support the assumption that ppGpp coordinates the expression of cellular factors required during an UTI process.

## 2. Results and Discussion

### 2.1. The Production of the α-Hemolysin Is Impaired in ppGpp-Deficient Strains

**Figure 1:**
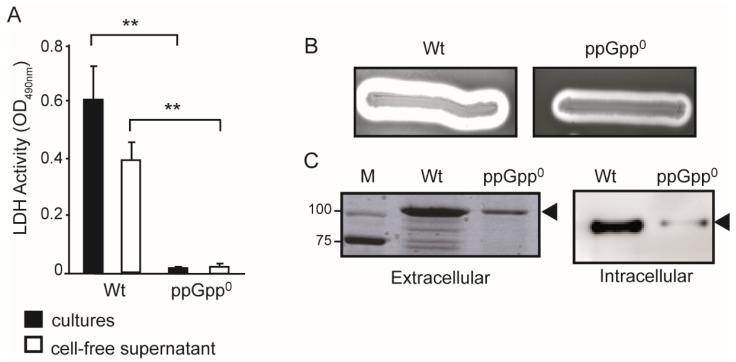
Effect of ppGpp on the expression of the *hlyCABD_II_* operon of J96. (**A**) Bacterial-induced cytotoxicity on T24 cells was monitored as lactate dehydrogenase (LDH) activity released (OD_490nm_) after 3 h incubation with bacterial cell suspensions (black bars) or cell-free supernatants (white bars). Bacterial cultures of the K-12 derivative AAG1 with plasmid pSF4000 (pACYC184 carrying *hlyCABD_II_*) and its ppGpp^0^ derivative (JFV2) were grown in LB at 37 °C up to late exponential phase (OD_600nm_ of 0.8). Data shown are mean and standard deviation of four independent determinations. Statistical significance levels are reported as ** = *p* < 0.005. (**B**) Hemolytic phenotype on blood agar plates of the AAG1 (Wt) and JFV2 (ppGpp^0^) carrying the plasmid pSF4000. (**C**) Amount of α-hemolysin produced in bacterial cultures of the same strains as in panel (**B**), grown in LB at 37 °C up to an OD_600nm_ of 0.8. Left panel, secreted α-hemolysin (extracellular) was detected in cell-free supernatant from the cultures by Coomassie blue stained 10% SDS-PAGE. Lane M: molecular mass markers (size in kDa indicated along the left side). Right panel, intracellular α-hemolysin was determined by immunoblot analysis. The band corresponding to the α-hemolysin is indicated by an arrowhead.

The effect of ppGpp on the expression of α-hemolysin produced by the *hlyCABD_II_* of J96 was initially characterized using the plasmid pSF4000, a pACYC184-based plasmid carrying the *hlyCABD_II_* operon. The pSF4000 plasmid was transformed in the *E. coli* K12 strain AAG1 (MG1655 Δlac) and its ppGpp-deficient derivative (ppGpp^0^) counterpart (JFV2). An increase in cytotoxicity by the presence of plasmid pSF4000 in a non-hemolytic strain has been previously described [22]. Infection challenges with both Wt and its ppGpp^0^ derivative carrying the pSF4000 plasmid were performed on T24 cells. Cytotoxicity, monitored as lactate dehydrogenase activity released from the T24 cells, was significantly higher in the Wt strain as compared to its isogenic ppGpp^0^ derivative (Figure 1A, black bars). A similar pattern was detected when T24 cells monolayers were incubated with cell-free supernatants from cultures of both strains (Figure 1A, white bars), confirming the involvement of a secreted product in the observed cytotoxic effect. The activity of the α-hemolysin can be easily characterized by its ability to lyse erythrocytes. Comparison of the hemolytic haloes surrounding the colonies on blood agar plates also substantiates the impaired ability to produce the toxin by the ppGpp-deficient strain (Figure 1B).

To further corroborate these results, the amount of secreted toxin was monitored in cell-free supernatants from cultures of both Wt and ppGpp^0^ strains. The α-hemolysin, with an estimated molecular mass of 110 kDa, was detected as a major band in cell-free supernatant of Wt cultures after SDS-PAGE and Coomassie staining (Figure 1C, labelled with an arrowhead). A similar band with much lower intensity was detected in the supernatant of the ppGpp^0^ strain. The amount of secreted α-hemolysin was about 8-fold lower in ppGpp^0^ as compared to Wt.

Our data show that ppGpp is required for optimal production of external α-hemolysin. This conclusion can be draw either by ppGpp acting as a positive regulator of the α-hemolysin expression or by ppGpp promoting its secretion to the external medium. If ppGpp is involved in the secretion of the α-hemolysin, one would expect accumulation of the toxin in the cytoplasm of the ppGpp^0^ strains. The intracellular α-hemolysin in whole cell extracts of Wt and ppGpp-deficient strains was monitored by immunoblot analyses (Figure 1C). The amount of intracellular α-hemolysin was higher (10-fold) in the Wt strain than in its ppGpp^0^ derivative, clearly indicating that ppGpp stimulates the production of α-hemolysin.

### 2.2. Suppressor Mutants of the Auxotrophic Phenotype Depicted by the ppGpp^0^ Restore α-Hemolysin Expression from the hlyCABD_II_ Operon

**Figure 2:**
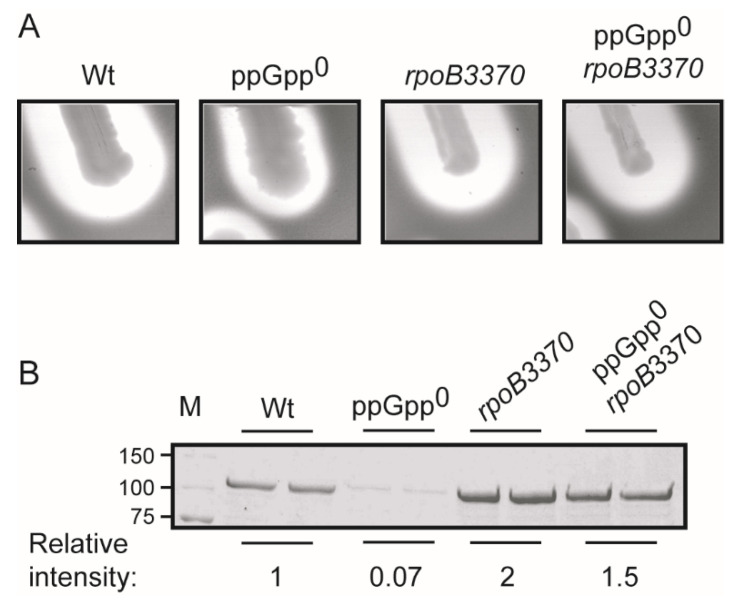
Analysis of the hemolytic phenotype of the ppGpp^0^ suppressor mutant *rpoB3370*. (**A**) Hemolytic phenotype on blood agar plates of Wt (AAG1), ppGpp^0^ (JFV2), *rpoB3370* (JFV19), and ppGpp^0^ *rpoB3370* (JFV20) carrying the plasmid pSF4000. (**B**) Electrophoretic analyses (Coomassie blue stained 10% SDS-PAGE) of the cell-free supernatants from bacterial cultures grown in LB at 37 °C up to late exponential phase (OD_600nm_ of 0.8) of the same strains as in (**A**). Relative intensities of the protein bands are indicated at the bottom of the panel. Lane M: molecular mass markers (size in kDa indicated along the left side).

The ppGpp^0^ *E. coli* strains are impaired to induce the expression of several amino acid biosynthetic pathways and, consequently, these strains are unable to grow on minimal media [23]. Spontaneous suppressor mutants of ppGpp^0^ strains restoring prototrophy can be selected by plating cultures on minimal media plates. Consistent with the fact that ppGpp binds to the RNA polymerase, mutations associated to prototrophy restoration are frequently located within the *rpoB* and *rpoC* genes that encode the β and β′ subunits of the RNA polymerase, respectively [23]. The *rpoB3370* allele carries a missense mutation causing the change in the amino acid 563 of the β subunit of the RNA polymerase from a threonine to a proline (T563P). The *rpoB3370* allele restores ppGpp-deficiency phenotypes [11,23,24]. The effect of *rpoB3370* on α-hemolysin expression from the *hlyCABD_II_* operon was scored. The α-hemolysin expression in the ppGpp-deficient derivative strain carrying the *rpoB3370* allele was restored to the same level as in the Wt strain (Figure 2).

Moreover, spontaneous prototrophic mutants from the ppGpp-deficient strain were selected in minimal media plates and the production of α-hemolysin was monitored on blood agar plates for 38 independent mutants. Up to 76% of the mutants restore α-hemolysin expression to levels detected in the ppGpp-proficient strain. The genetic link between the α-hemolysin expression and suppressor mutations of the ppGpp-deficiency phenotype further confirm the involvement of ppGpp in the regulation of α-hemolysin expression.

### 2.3. ppGpp Stimulates the Transcriptional Expression of the hlyCABD_II_ Operon

**Figure 3:**
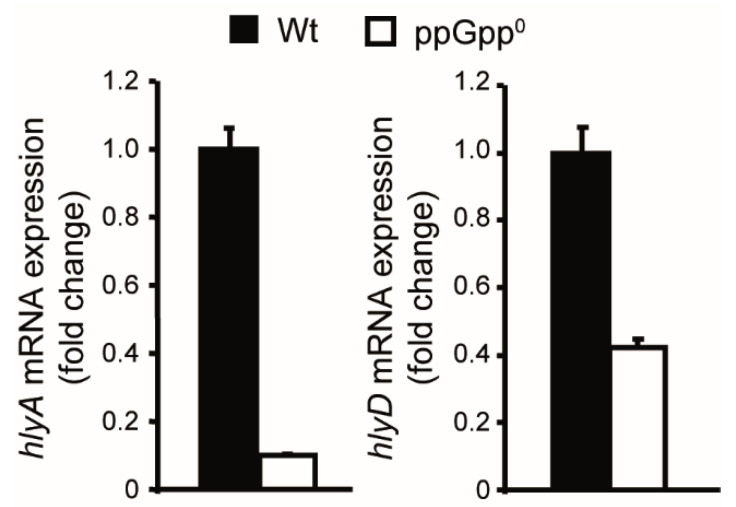
ppGpp stimulates the transcriptional expression of the *hlyCABD_II_* operon from J96. Relative *hlyA* and *hlyD* mRNA quantification by qPCR in Wt (AAG1) and ppGpp^0^ (JFV23) strains carrying plasmid pMGP-1 (pBR322 based plasmid carrying the *hlyCABD_II_* operon of J96). RNA samples were extracted from cultures grown in LB at 37 °C up to late exponential phase (OD_600nm_ of 0.8). The results are mean and the standard deviation from three biological repeats.

Transcription from the *hlyCABD* operons generates two transcripts, *hlyCA* and *hlyCABD*, through transcriptional termination occurring between *hlyA* and *hlyB* [25]. Transcriptional studies by qPCR were performed to determine the levels of *hlyA* and *hlyD* transcripts from the *hlyCABD_II_* operon (Figure 3). An obvious reduction for both transcripts was detected in the ppGpp^0^ strain as compared to Wt, being more pronounced for the *hlyA* transcript. These results indicate that ppGpp stimulates the transcriptional expression of the *hlyCABD_II_* operon. The differential effect of ppGpp on the expression of *hlyA* and *hlyD* suggest that ppGpp affects somehow the termination occurring in the internal terminator.

### 2.4. The ppGpp-Mediated Regulation of the hlyCABD_II_ Operon Is Independent of RpoS, RfaH, Zur, and H-NS

**Figure 4:**
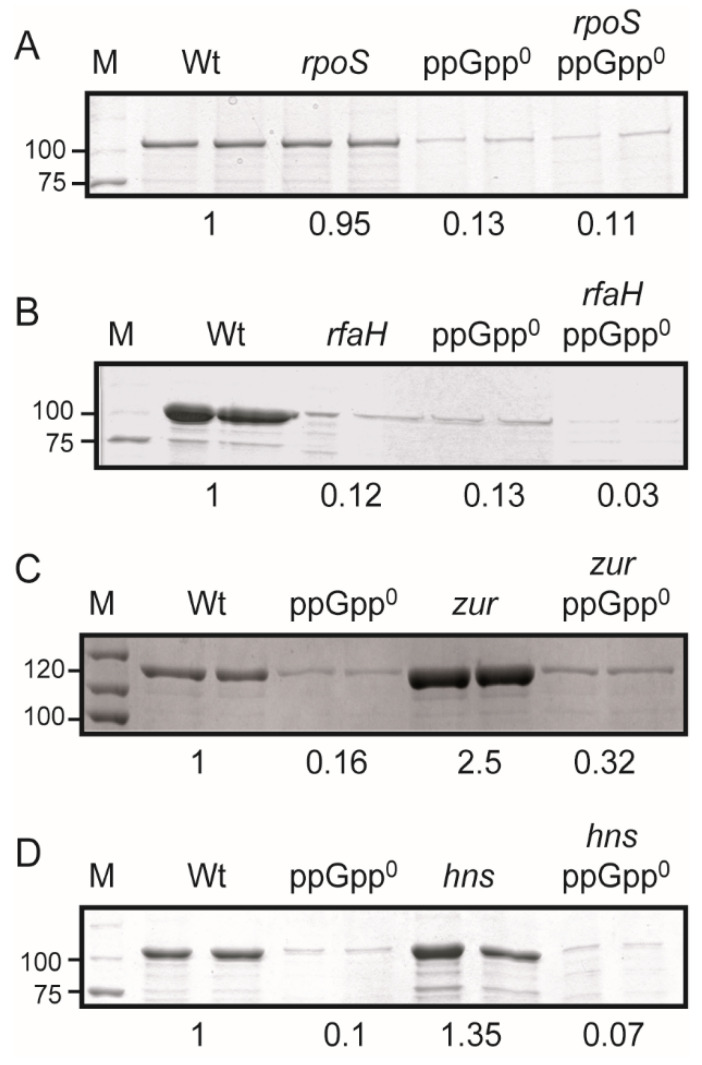
ppGpp-mediated stimulation of *hlyCABD_II_* expression is independent of RpoS, RfaH, and H-NS. Electrophoretic analyses (Coomassie blue stained 10% SDS-PAGE) of cell-free bacterial culture supernatants of ppGpp-proficient and ppGpp-deficient derivatives of *rpoS* (**A**), *rfaH* (**B**), *zur* (**C**), and *hns* (**D**) and its corresponding Wt counterparts (AAG1/pSF4000 for *rpoS* and *hns* analyses and AAG1/pMGP-1 for *rfaH* and *zur* analyses). Cultures were grown in LB at 37 °C up to late exponential phase (OD_600nm_ of 0.8). Lane M: molecular mass markers (size in kDa indicated along the left side). The relative average intensity of the bands is indicated at the bottom of the panel.

ppGpp promotes the expression of RpoS-dependent genes by stimulating both *rpoS* expression and the interaction of RpoS with the RNA polymerase holoenzyme [2]. RpoS-deficiency did not cause any significant reduction in the α-hemolysin expression from the *hlyCABD_II_* operon as compared to the Wt strain. Moreover, the absence of ppGpp causes a reduction in the production of α-hemolysin in both *rpoS* and *rpoS*^+^ strains (Figure 4A), indicating that *hlyCABD_II_* expression is RpoS-independent.

The transcriptional antiterminator RfaH regulates *hlyCABD_II_* expression [26]. Hence, *rfaH* mutation causes a dramatic reduction in the expression of the α-hemolysin (Figure 4B). Nevertheless, the *rfaH* strain was still responding to ppGpp since a significant reduction was observed in the ppGpp-deficient strain when *rfaH* was removed. To corroborate the accumulative effect of ppGpp^0^ and the *rfaH* mutation, *hlyA* mRNA levels were determined by qPCR. Consistent with previously published data [27], a clear reduction in the level of *hlyA* transcript was observed in the *rfaH* mutant strain (5-fold reduction). A further decrease was observed in a ppGpp-deficient genetic background, consistent with the results at the protein level (Figure S1, Supplementary Materials). These results suggest that the ppGpp-mediated regulation of the *hlyCABD_II_* operon is also independent of RfaH.

Zur, a regulator that senses the intracellular levels of zinc in the cell [28], represses the *hlyCABD*_II_ operon. The derepressed expression of the α-hemolysin by the lack of Zur repressor, observed previously in the J96 strain, was also detected in the K12 genetic background (Figure 4C). ppGpp deficiency caused a drastic drop in the α-hemolysin expression even in the absence of the Zur repressor. These results suggest that Zur is not involved in the ppGpp-mediated regulation of the *hlyCABD_II_* operon.

The H-NS protein has been found to silence the expression of the α-hemolysin encoded in the *hlyCABD* operon from the conjugative plasmid pHly152 [29]. To study the H-NS effect in the *hlyCABD_II_* operon, the *hns* allele was introduced in Wt and ppGpp-deficient derivatives (Figure 4D). Under the experimental conditions used, only a slight increase in the α-hemolysin expression level was detected in the *hns* mutant as compared with the isogenic Wt counterpart. Similar results were obtained when characterizing the effect of the *hns* mutation in the UPEC strain 536 [30]. Nonetheless, the decrease in the α-hemolysin expression caused by the ppGpp deficiency was independent of the presence or absence of H-NS, indicating that H-NS is not involved in the ppGpp-dependent regulation of the *hlyCABD_II_* operon.

Overall, our data indicate that the ppGpp-mediated stimulation of the *hlyCABD_II_* operon is independent of RpoS and the previously described α-hemolysin regulators RfaH, Zur, and H-NS. Further studies will be required to fully dissect the exact mechanism by which ppGpp mediates the stimulation of *hlyCABD_II_* operon expression.

### 2.5. ppGpp Is Required for the Thermoregulation but Not for the Osmoregulation of hlyCABD_II_ Expression

The expression of the α-hemolysin in the conjugative plasmid pHly152 is affected by environmental parameters such as temperature and osmolarity of the culture media [31]. Experiments were performed to determine whether the expression of the *hlyCABD_II_* operon was equally thermo- and osmo-regulated, and to describe a possible role of ppGpp in the environmental regulation.

**Figure 5:**
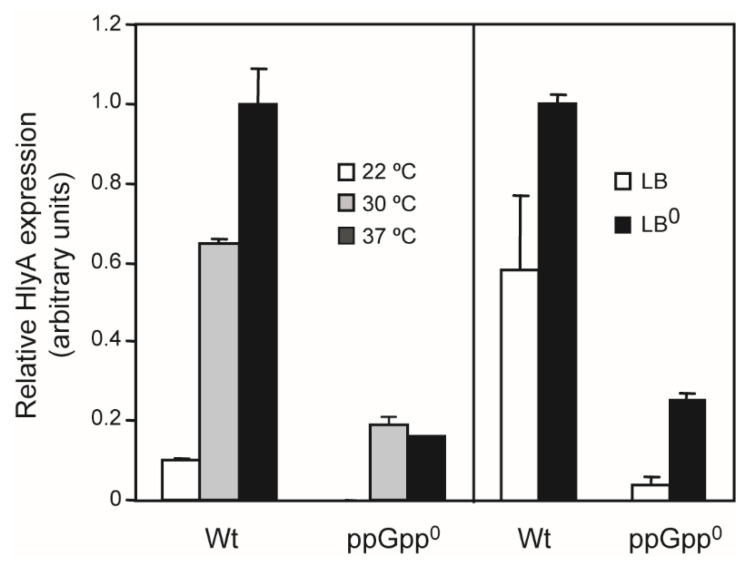
Thermo- and osmo-regulation of the *hlyCABD_II_* operon. Cultures of the Wt (AAG1) and ppGpp^0^ (JFV2) carrying the plasmid pSF4000 were grown up to an OD_600nm_ of 0.8 either in LB at 22, 30, and 37 °C (left panel) or at 37 °C in either LB (170 mM NaCl) or LB^0^ (0 M NaCl) (right panel). The protein content of the cell-free supernatants was analyzed (10% SDS-PAGE and Coomassie blue stained) and the relative amount of α-hemolysin is plotted in the bar diagram. After band intensity quantification, a value of 1.0 was arbitrarily given to the amount of α-hemolysin detected in cultures of the Wt strain in LB at 37 °C. Data shown are mean and standard deviation of toxin determination from two independent cultures.

As seen in Figure 5, α-hemolysin expression, monitored as the amount of HlyA present in the external fraction, is regulated by both temperature and external osmolarity. The toxin was barely detectable in cultures grown at low temperature (22 °C), a significant increase was detected when growing at 30 °C, and the maximal expression was detected at 37 °C (Figure 5, left panel). The ppGpp-deficient strain did not grow at 22 °C, even after a prolonged incubation period. At 30 °C, the amount of α-hemolysin was significantly lower compared to the Wt strain, and no further induction was observed when the incubation temperature was raised to 37 °C (Figure 5, left panel). Regarding the osmolarity, the α-hemolysin expression is induced at low osmolarity (LB with 0 M NaCl) in both ppGpp-proficient and -deficient strains (Figure 5, right panel). These data indicate that ppGpp is required for the thermoregulation of α-hemolysin expression, but not for its osmoregulation.

### 2.6. ppGpp-Deficiency Causes a Drastic Drop in the α-Hemolysin Production in the Uropathogenic Isolate J96

**Figure 6:**
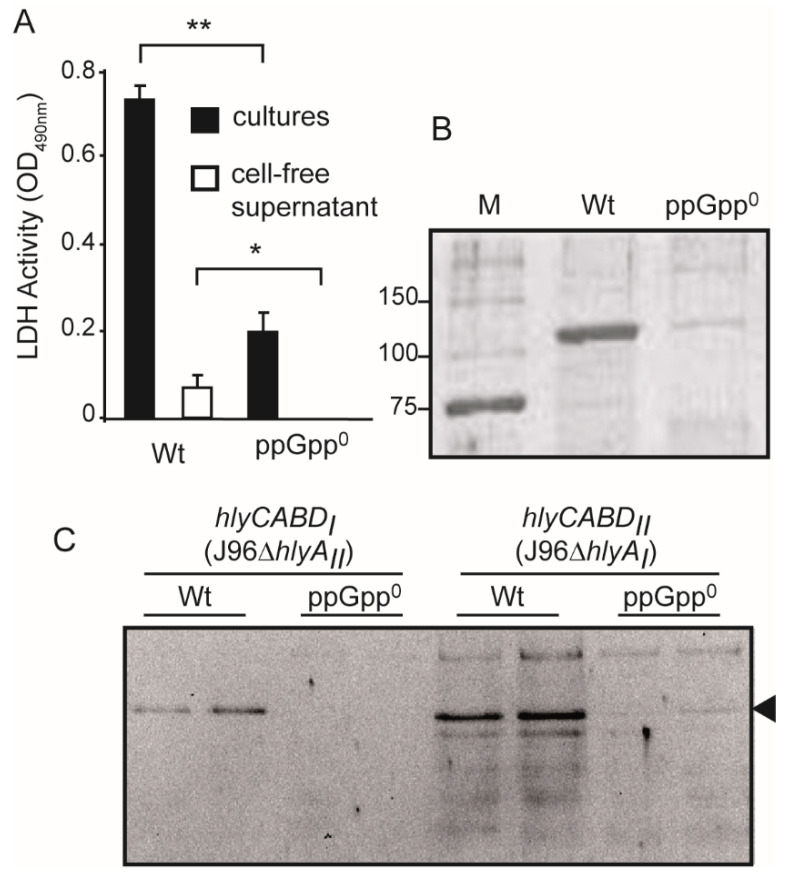
The UPEC-induced cytotoxicity on bladder epithelial cells is reduced in ppGpp-deficient derivatives by affecting production of α-hemolysin. (**A**) Bacterial-induced cytotoxicity on T24 bladder epithelial cells was monitored as lactate dehydrogenase (LDH) activity released (OD_490nm_) after 3 h incubation with bacterial cultures (black bars) or cell-free supernatants (white bars). Bacterial cultures of the UPEC isolate J96 and its ppGpp-deficient derivative (J96 r*elA spoT*, ppGpp^0^) were grown in LB at 37 °C up to late exponential phase (OD_600nm_ of 0.8). The results are mean and standard deviation of four independent determinations. Statistical significance levels are reported as: * = *p* < 0.05; ** = *p* < 0.005. (**B**) Coomassie blue stained 12.5% SDS-PAGE analyses of cell-free supernatants from cultures used in A. Lane M: molecular mass markers (size in kDa indicated along the left side). (**C**) The expression of the two hemolytic operons, *hlyCABD_I_* and *hlyCABD_II_*, present in the J96 strain is stimulated by ppGpp. Immunodetection of the secreted α-hemolysin from cell-free supernatants of cultures of Wt and ppGpp^0^ derivatives of J96Δ*hlyA_II_* (JFV16 and JFV18) and J96Δ*hlyA_I_* (JFV21 and JFV22) grown in LB at 37 °C up to an OD_600nm_ of 0.8. The band corresponding to the α-hemolysin is indicated by an arrowhead.

As previously indicated, the *hlyCABD_II_* operon is located in a pathogenicity island of the pyelonephritis isolate J96 [17,18]. Experiments were conducted to confirm that the ppGpp regulation described also occurs in the natural genetic background of the *hlyCABD_II_* operon. Cytotoxicity assays on T24 cells were performed with LB cultures from J96 and its ppGpp-deficient derivative and cell-free supernatants from these cultures (Figure 6A). The data obtained indicate that the cytotoxicity is severely impaired in the ppGpp^0^ mutant derivative compared to Wt. Moreover, the amount of secreted α-hemolysin was about 10-fold lower in ppGpp^0^ compared to Wt in cell-free supernatants, as seen by Coomassie staining, after SDS-PAGE (Figure 6B).

The J96 strain carries two *hlyCABD* operons, the extensively studied *hlyCABD_II_* operon, and the *hlyCABD_I_* operon, which has been poorly characterized. The coding sequences from both operons are almost identical, whereas the sequences upstream of *hlyC* carrying the promoter and regulatory motifs clearly diverge between both operons [28]. The two operons respond differently to specific environmental inputs, suggesting a specific role at distinct stages during the infection process [18,28]. The expression from the two *hlyCABD* operons present in the strain J96 was studied, using deletion mutants of the *hlyA_I_* and *hlyA_II_* genes, and monitoring the amount of external toxin produced in both Wt and ppGpp^0^ strains (Figure 6).

ppGpp is required for the appropriate expression of the *hlyA* gene from both *hlyCABD* operons of the J96 strain. A reduction in the production of α-hemolysin from both hemolytic operons was detected in the corresponding ppGpp^0^ derivatives. Despite the disparity of the regulatory regions, the expression of the α-hemolysin from both operons is stimulated by ppGpp, indicating a central role for this second messenger in the expression of this toxin. Interestingly, under the culture conditions used, there is apparently higher production of α-hemolysin from the *hlyCABD_II_* operon. The downregulation of the hemolysin production from the *hlyCABD_II_* operon in both commensal and uropathogenic isolates clearly indicates that no UPEC specific factor is strictly required for the ppGpp-mediated regulation described.

## 3. Conclusions 

An infectious process requires a constant adaptation of the bacteria to the different environments encountered within the host. During this adaptive response, bacteria undergo severe reprogramming that affects genes from virtually all the functional categories. In many pathogens, genes that encode functions directly implicated in the pathogenesis have been acquired by horizontal gene transfer (HGT) mechanisms [32].

An intense interplay among core genome-encoded regulators and HGT-genetic systems exists. Most studies have focused on silencing systems, mediated by core genome-encoded regulators that suppress uncontrolled expression of newly arrived genetic systems to promote establishment of the new genes in the genome. These mechanisms are exemplified by the H-NS-mediated silencing of HGT-DNA described in different bacterial species [33]. Here, we described a core genome-encoded regulator, ppGpp, that stimulates the expression of a genetic system that has been acquired by HGT, the *hlyCABD_II_* operon. This interplay ensures a positive stimulation of the expression of the newly acquired genes when required, thereby highlighting the importance of the responsiveness to stress sensors in the expression of colonization and/or virulence factors. The ppGpp-mediated stimulation of HGT gene systems related with virulence has also been described in other microorganisms such as *Salmonella*, where most of the genes present in the pathogenicity islands are under the control of ppGpp [9,34,35]. Our results suggest that, during the course of an infection, when bacteria encounter environmental and/or nutritional stress, the associated increase in the intracellular level of ppGpp causes a concomitant change in the gene expression pattern, reprogramming the bacterial metabolism, physiology, and pathogenicity, to promote survival of bacteria inside the host.

## 4. Materials and Methods

### 4.1. Bacterial Strains, Plasmids, and Growth Conditions

Bacterial strains and plasmids used in this work are shown in Table 1. All strains were grown routinely in Luria-Bertani (LB; 10 g/L tryptone, 10 g/L NaCl, 5 g/L yeast extract, CONDA, Torrejon de Ardoz, Spain) broth or agar (15 g/L agar) at 37 °C unless otherwise noted. To monitor hemolytic phenotype, blood agar plates were used (LB agar plates supplemented with 5% sheep blood). For growing cultures under low-osmolarity conditions, LB without NaCl was used and denoted LB^0^. Bacterial growth was monitored by measuring the OD_600nm_ in a Beckman Coulter^TM^ DU^®^ 640 spectrophotometer (Beckman Coulter, Pasadena, CA, USA) where 0.5 units of OD_600nm_ correspond to mid-log phase growth and approximately 5·10^8^ bacteria/mL. All ppGpp-deficient strains were tested for auxotrophy phenotype on minimal media plates with the following composition: 1× M9 salts, 2 mM MgSO_4_, 0.1 mM CaCl_2_, 0.4% glucose, 0.02 mM thiamine, and 1.5% bactoagar. When required, antibiotics were added at the following concentrations: kanamycin (km) 25 μg mL^−1^, tetracycline (Tc) 15 μg mL^−1^, chloramphenicol (cm) 15 μg mL^−1^, and ampicillin (Ap) 50 μg mL^−1^.

### 4.2. Genetic Techniques

The primers used in this work are listed in Table S1 (Supplementary Materials). Different gene alleles were transferred by P1 transduction using the following donor strains: CF1693 for *relA251::Km*^R^ and *spoT207::Cm*^R^, RH90 for *rpoS359::Tn10*, EC3954 for *rpoB3370 thi::Tn10*, BSN27 for Δ*hns-trp::Tn10*, 5KC 1.8 for *rfaH::Tn5 1.8*, and MG1655 Δ*zur* for Δ*zur::Cm^R^*. The deletion mutant strain MG1655 Δ*zur* was generated using the primer pair zurP1/zurP2 and the method described by Datsenko and Wanner [36]. The strain AAG16 carries a *relA* disruption from codon 6 to 743, obtained as described by [20]. The plasmid pMGP-1 carries the same fragment as in pSF4000 in pBR322. In all studies undertaken, cells carrying either pSF4000 or pGMP-1 showed apparently identical phenotypes.

**Table 1:** Strains and plasmids used in this work.

Strain	Relevant Characteristics	Reference
J96	Pathogenic isolate	[37]
J96 *relA spoT*	J96 *relA251*::Km^R^, *spoT207*::Cm^R^	[19]
JFV16	J96 Δ*hlyA_II_*	[28]
JFV18	JFV16 *relA251*::Km^R^, *spoT207*::Cm^R^	This study
JFV21	J96 Δ*hlyA_I_*	[28]
JFV22	JFV21 *relA251*::Km^R^, *spoT207*::Cm^R^	This study
AAG1	MG1655 Δ*lacZ*	[38]
CF1693	MG1655 *relA251*::Km^R^, *spoT207*::Cm^R^	[39]
JFV2	AAG1 Δ*relA* Δ*spoT*	[20]
JFV23	AAG16 *spoT207*::Cm^R^	This study
EC3954	MG1655 *rpoB3370 thi*::Tn10	[19]
JFV19	AAG1 *rpoB3370 thi*::Tn10	This study
JFV20	JFV2 *rpoB3370 thi*::Tn10	This study
RH90	MC4100 *rpoS359*::Tn10	[40]
AAG18	AAG1 *rpoS359*::Tn10	This study
5KC 1.8	*recAI hsdR hsdS rfaH*::Tn5 1.8	[41]
JFV14	AAG1 *rfaH*::Tn5 1.8	This study
JFV15	JFV2 *rfaH*::Tn5 1.8	This study
BSN27	MC4100 Δ*hns-trp*::Tn10	[42]
JFV5	AAG1 Δ*hns-trp*::Tn10	This study
JFV6	JFV2 Δ*hns-trp*::Tn10	This study
MG1655Δ*zur*	MG1655 Δ*zur*::Cm^R^	This study
CJM1	AAG1 Δ*zur*::Cm^R^	This study
CJM2	JFV2 Δ*zur*::Cm^R^	This study
**Plasmid**		
pSF4000	*hlyCABD*_II J96_ in pACYC184, Cm^R^	[43]
pMGP-1	*hlyCABD*_II J96_ in pBR322, Ap^R^	This study

### 4.3. Cytotoxicity Assay

Cell cytotoxicity was assayed on semiconfluent monolayers of T24 bladder epithelial cells (3·10^4^ cells/well) cultured at 37 °C in 5% CO2 in RPMI-1640 media (Gibco, Thermo Fisher Scientific, Waltham, MA, USA), supplemented with 10% fetal bovine serum and 1% L-glutamine in 96-well tissue culture plates. The induced cytotoxicity by either bacterial strains or their cultures supernatants on T24 epithelial cells was monitored using the commercial kit Cytotox 96 (Promega corporation, Madison, WI, USA). Bacterial cultures were grown up to an OD_600nm_ of 0.8 in LB at 37 °C. Cells and supernatants were separated by centrifugation and the resulting supernatants were filtered (0.22 µm) to remove residual cells. The cytotoxicity of the secreted fraction was assessed by adding 25 µL of the cell-free supernatants into each well containing T24 cells covered by 100 µL of RPMI-1640 media. Infection was performed by adding 25 µL of LB bacterial suspension diluted to achieve a multiplicity of infection (MOI) of 10. After 3 h incubation, the plates were centrifuged (5 min at 400× *g*) and the cell culture supernatants were used to monitor the lactate dehydrogenase (LDH) release following the indications of the manufacturer. Results shown are the average and standard deviation of four replicas for each condition.

### 4.4. SDS-PAGE and Western Immunobloting Analyses

The standard SDS-PAGE procedure was used and gels were stained with Coomassie blue. For immunoblotting, proteins were transferred to a PVDF membrane and detected with the monoclonal anti-α-hemolysin H10 [44] and a horseradish peroxidase-conjugate antibody (Promega corporation, Madison, WI, USA) using the GE Healthcare^TM^ ECL Plus Western Blotting Detection System (GE Healthcare, Chicago, IL, USA). Gels were analyzed on a Chemidoc System (BioRad, Hercules, CA, USA) equipped with the QuantityOne^®^ Software for quantification.

### 4.5. Expression Analysis by qPCR

Total RNA samples were isolated from 1 mL of three independent cultures grown in LB at 37 °C up to an OD_600nm_ of 0.8 using the SV Total RNA Isolation System kit (Promega). Total RNA samples were treated with DNase (TURBO DNA-free ^TM^; Ambion, Thermo Fisher Scientific, Waltham, MA, USA). RNA quantity and quality was analyzed using the Bioanalyzer 2100 (Agilent Technologies, Santa Clara, CA, USA).

qPCR assays were performed using an ABI Prism 7700 sequence detector and TaqMan^®^ RNA-to-CTTM 1-Step kit (Applied Biosystems, Waltham, MA, USA). Total RNA samples (10 and 100 pg to quantify *hlyA* and *hlyD* mRNA levels, respectively) were run in triplicate. For control of DNA contamination, RT-PCR assay was performed, in parallel, with reverse transcriptase inactivated by heat-shock (95 °C for 15 min). Primers and probes (Table S1) were designed using the primer design software Primer Express (Applied Biosystems) and synthesized by Applied Biosystems.

### 4.6. Isolation of Suppressor Mutants

For the isolation of suppressor mutants, the strain JFV2 was grown in LB medium at 30 °C until mid-log phase. Cells were washed, plated on M9 minimal media plates, and incubated at 37 °C. ppGpp-deficient suppressor mutants were selected based on their ability to grow in minimal media (prototrophs). A collection of 38 clones was tested for their hemolytic phenotype on blood agar plates after introducing the pSF4000 plasmid.

### 4.7. Statistical Analysis

Differences between average values were tested for significance by performing an unpaired two-sided Student’s *t*-test. The levels of significance of the resulting *p*-values are indicated.

## Data Availability

Not applicable.

## Supplementary Materials

The supporting information can be downloaded at: https://www.mdpi.com/article/10.3390/ijms232012256/s1.
