# Supplementary material for: ppGpp, the General Stress Response Alarmone, Is Required for the Expression of the α-Hemolysin Toxin in the Uropathogenic Escherichia coli Isolate, J96
Source: Int J Mol Sci. 2022 Oct 14;23(20):12256. doi: 10.3390/ijms232012256 (PMC9602796; doi:10.3390/ijms232012256)
Supplement: Supplementary file 1 [file ijms-23-12256-s001.zip › ijms-1978498-supplementary.pdf]

Supplementary material

Manuscript:

ppGpp, the general stress response alarmone, is required for the expression of the α -hemolysin toxin in the uropathogenic *Escherichia coli* isolate, J96.

by Jorge Fernández-Vázquez, Juan David Cabrer-Panes, Anna Åberg, Antonio Juárez, Cristina Madrid, Tania Gaviria-Cantin, Llorenç Fernández- Coll, Andrés Felipe Vargas, Carlos Jonay Jiménez and Carlos Balsalobre

Table S1: Primers used in this study

Figure S1: Transcriptional expression of *hlyA* in strains AAG1 (Wt), JFV14 (*rfaH*), JFV23 ($ppGpp^0$) and JFV15 (*rfaH*$ppGpp^0$) carrying pMGP-1 by qPCR.

Table S1. Primers used in this work.

	**Sequence**
zurP1	CCATGTGCTTCAATAACATTATGCCGCAGGGCAAAACCCATTGT GTAGGCTGGAGCTTCG
zurP2	GAAAAGACCACAACGCAGGAGTTATTAGCGCAGGCTGAAACATA TGAATATCCTCCTTA
HlyA up	GATTTCCGGGATGTGGCC
HlyA Taqman probe	TCAGGCAGAAAGGT
HlyA down	CCTTCAGCTTTATACATGATGAGGTC
HlyD up	ACATTCCATTAAGCTCGGGTATG
HlyD Taqman probe	CTGTCACTGCAGAAAT
HlyD down	ACGCTTCGCATTCCAGTCTT

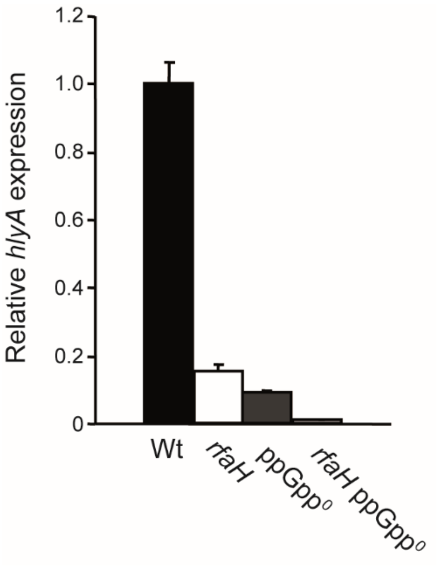

Figure S1: Transcriptional expression of *hlyA* in strains AAG1 (Wt), JFV14 (*rfaH*), JFV23 (ppGpp^0) and JFV15 (*rfaH*ppGpp^0) carrying pMGP-1 by qPCR. RNA samples from cultures grown in LB at 37ºC up to an OD_{600nm} of 0.8. The results are the arithmetic mean and the standard deviation from three biological repeats.
